# Home Sweet Home: New Insights Into the Location of Equine Premises in France and Keeping Habits to Inform Health Prevention and Disease Surveillance

**DOI:** 10.3389/fvets.2021.701749

**Published:** 2021-08-23

**Authors:** Halifa Farchati, Aurelie Merlin, Mathilde Saussac, Xavier Dornier, Mathilde Dhollande, David Garon, Jackie Tapprest, Carole Sala

**Affiliations:** ^1^Laboratory for Animal Health in Normandy, Physiopathology and Epidemiology of Equine Diseases Unit, French Agency for Food, Environmental and Occupational Health & Safety (ANSES), Goustranville, France; ^2^University of Lyon - Epidemiology and Support to Surveillance Unit, ANSES, Lyon, France; ^3^Normandie Univ, UNICAEN, ABTE, Caen, France; ^4^French Horse and Riding Institute (IFCE), Pompadour, France

**Keywords:** equine, keeper, owner, holding, traceability, spatial location, epidemiological surveillance, health monitoring

## Abstract

Identifying and tracking equines are key activities in equine health prevention. France is one of the few European countries with an operational centralized database that records information on equines, owners, and keepers but not on the location and keeping conditions of equines. The objective of our study was to collect information on keeping habits of equines and the relative location of a wide range of equines, owners, and keepers and discuss their implication for surveillance and control of outbreak improvement. A national email survey was conducted among the 1.9% of people registered as owners and 8.2% of people registered as keepers in the French national equine identification database having given their agreement to be contacted by email. It led to the collection of information from 728 owners, 121 keepers, and 2,669 owner–keepers. Most of them housed their equines in a single *commune* (smallest geographic administrative unit in France) at their home as private individuals. The distance between the *communes* of residence and of holding was, in most cases (including 79% of owners in the owner survey, 89.5% of the keepers in the keeper survey, and about 94% of the owner–keepers in both surveys), less than 30 km. More than half of the keepers kept a maximum of five equines and the majority with two different uses/destinations together, mostly leisure-retirement, leisure-breeding, leisure-sport, and sport-breeding. The main limitation of the study was that a relatively limited number of people (*n* = 3518) were reachable due to the low availability of an email address and contact agreement. Nonetheless, the findings provide an overview of how equines are kept by non-professional owners and keepers and complements information usually collected by the French riding institute. Additionally, information collected is very helpful to determine a realistic estimate of the spatial distribution of equines in France. This information is very important for the equine sector, for demographic knowledge and also improvement of surveillance plans and control measures and for the management and monitoring of health events to limit the spread of diseases.

## Introduction

Over the past 20 years, significant efforts have been made to identify and track equines in France. However, regarding location, a major traceability problem persists. In fact, although current regulations in France require keepers to declare the equines they keep in a breeding register, this information is not available in the centralized SIRE database. This makes it difficult to manage and monitor health events, especially epidemics, and also creates an issue in research when a better understanding of the spread of a disease is needed. In France, equine traceability is based on the centralized SIRE database (equine information system), managed by the French Horse and Riding Institute (IFCE), which registers almost all equines born in or imported into France. The SIRE database includes, in the “equine-owner” data set, individual information on equines, including SIRE number, sex, breed, and date of birth, and information concerning the declared owner, such as the SIRE identification number and code (unique identification number) of the *commune* of the owner's residence. In parallel, in the “equine-premises” data set, there is information related to keepers as well as their premises: the identification number of the keeper, the *commune* code for the keeper's residence and those of the premises, and the opening date of the premises. However, at this time, there is no specific traceable link in the SIRE database between keepers and the equines they keep and, therefore, a lack of centralized information in France. The lacking information includes equine location even though this is a major concern for surveillance and control of outbreaks. Each year, the Economics Department of the IFCE collects information from all stakeholders in the horse industry to provide key, up-to-date figures on equine sector activities and equine socioeconomic statistics (1). However, the information collected mostly concerns professionals (breeders, horse trainers, barn managers, etc.) and the number of equines kept in professional structures (2). Data on non-professional owners and keepers are difficult to obtain even though this concerns one third of equines housed in France and may play a key role in equine disease transmission. Within this context, two national surveys were conducted in 2019 in collaboration with the IFCE with a dual objective: to evaluate the quality of information recorded in the SIRE database (3) and to collect information on equine premises (use/destination and location) and keeping habits of owners and keepers that escape regular IFCE data collection.

## Materials and Methods

### Sample

**Figure 1:**
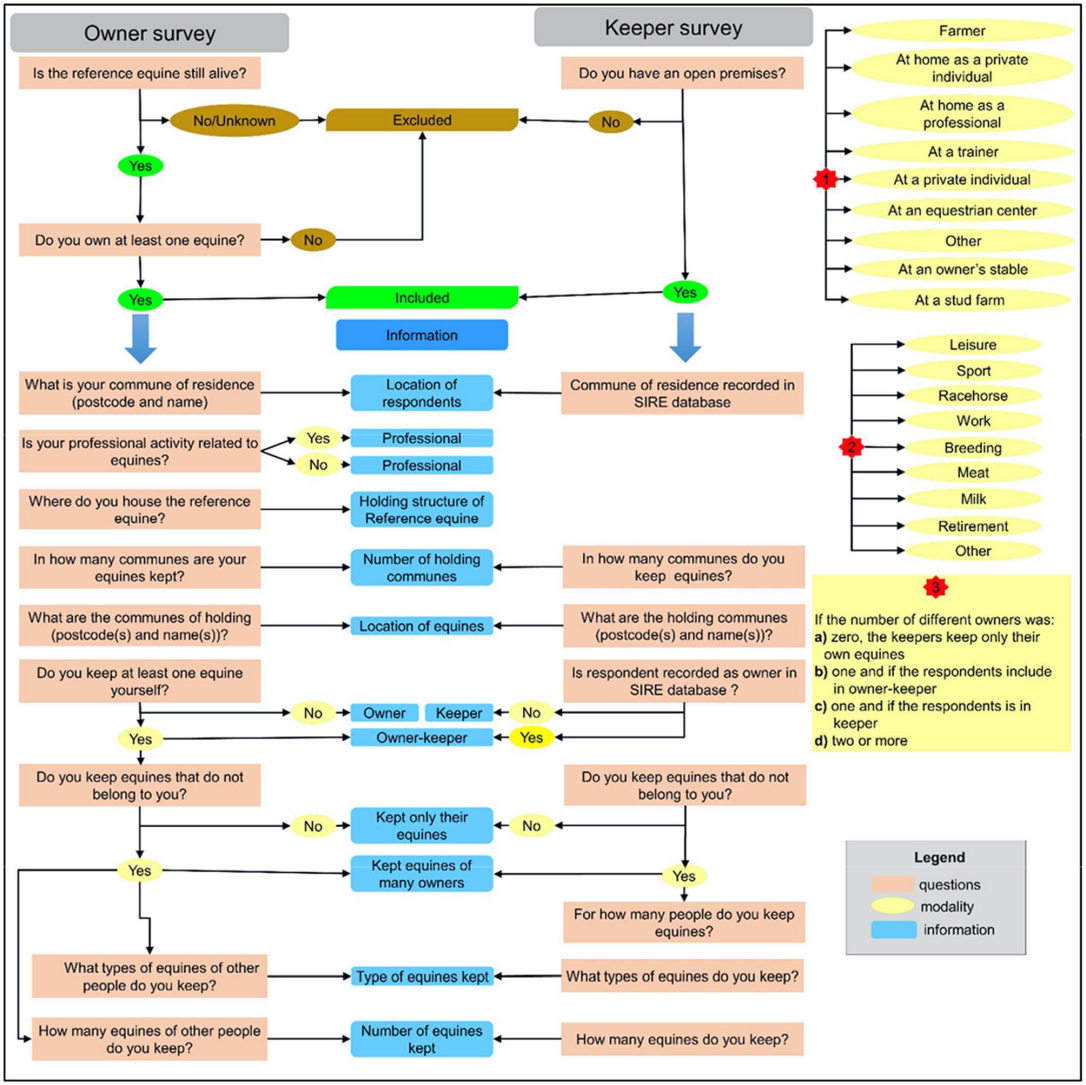
Structure of questionnaires, information, and decision rules.

We used a subsample of the surveys described by Farchati et al. (3). Briefly, these surveys were conducted among the 1.9% of the 737,789 owners and 8.2% of the 76,501 keepers registered in the SIRE database who had an available email address and agreed to be contacted in this way. Owners and keepers were surveyed differently because different information was available in the database (3) (Figure 1). The owner survey focused on information on owners and all their equines. To facilitate answers to the questionnaire, for each owner, a series of questions focused on one equine (*reference equine*, later in the text), randomly selected among the living equines, for the owners linked to several equines, or corresponding to the one that died most recently for owners with only dead equines in the SIRE database. The keeper survey concerned keepers having declared at least one premises and aimed to characterize keepers and equine premises.

We included in the current study the 3,518 respondents declaring at the time of the survey that they owned and/or kept at least one equine: 2,377 from the owner survey and 1,141 from the keeper survey, including the 429 people who responded to both surveys. When questions differed in the surveys, these 429 persons were included in both surveys (Figure 1). For similar questions, we kept only responses from one questionnaire after checking for consistency of the responses in the two surveys (Figure 1).

### Gathered Data

The information collected concerned respondent characterization: owner and/or keeper, professional activity related to the equine sector and location, equine keeping habits (holding structures, number and use/destination of equines kept) (3), and the location of equines (postcode and names of the *commune*). Details on questions, their correspondence between surveys, and their interpretation are presented in Figure 1.

### Additional Information

Additional information was used to interpret and discuss results. The keeper locations (a question not asked in the keeper survey) were, thus, extracted from the SIRE database. The CORINE Land Cover (CLC) geographic database (a biophysical inventory of European land use and its evolution according to a 44-item nomenclature) (4) was used to explore the effect of urban coverage on the distance between owners/keepers and the equines. For the calculation of the proportion of urban coverage, we grouped the percentage of continuous and discontinuous urban tissue supplied in the third level of the nomenclature of the CLC.

### Analysis

We analyzed the information by respondent status to compare the habits of the following:

- *Owner–keepers* (from both surveys): respondents recorded as owners in the SIRE database and who reported keeping at least one equine (owner survey) and respondents recorded as keepers in the SIRE database and who reported owning at least one equine;
- *Owners*: respondents who reported owning at least one equine but not keeping any equines, even their own;
- *Keepers*: respondents who reported keeping at least one equine but not owning any equines.

Location information provided by respondents (postcode and/or the name of the *commune* of holding and/or residence) was cross-checked with the French National Institute of Statistics and Economic Studies (INSEE) database to obtain the unique INSEE code for each *commune*. This information was used to calculate the distance between the *commune* of residence and the *commune* of holding as the distance between the centroid of the *communes*. The “gCentroid” and “gDistance” functions of R software were used to identify the centroid of each *commune* and to calculate the distance between the centroids of the *communes*. The surface area of *communes* in mainland France is highly variable, between 0.03 and 757.77 km^2^, with a mean of 17.71 km^2^ [median = 12.19 km^2^, standard deviation = 19.92 km^2^]. The mean distance between the centroids of neighboring *communes* is 4.91 km [min = 0.17 km, max = 34.02 km, median = 4.47 km, and standard deviation = 2.27 km].

Data processing and analysis were performed with R Studio interface software, version 3.6.1 (5). Associations between two qualitative variables were tested using Pearson's Chi-square test, and a Wilcoxon's or Kruskal–Wallis test was used to compare the means of two distributions with an error threshold set at 0.05. A correlation test was done to assess an association (dependence) between the distances of equines from their owners and/or keepers and urban coverage.

## Results

### Characterization of Respondents

Among the 2,377 owners selected for the owner survey, 69% (*n* = 1649) were owner–keepers, and 31% (*n* = 728) were strictly owners. Among the 1,141 keepers selected from the keeper survey, 89% (*n* = 1020) were owner–keepers, and 11% (*n* = 121) were strictly keepers (Table 1). Finally, our result concerned 728 owners, 121 keepers, and 2,669 owner–keepers (including the 429 owner–keepers who responded to both surveys) as defined in methodology section.

**Table 1:** Major characteristics of respondents by type of respondent (owner, owner–keeper, or keeper, professional activity and holding duration) and distance between respondent and equines locations.

		**Owner survey (** ***n*** **=** **2377)**				**Keeper survey (** ***n*** **=** **1141)**			
		**Owner (** ***n*** **=** **728)**		**Owner-keeper (** ***n*** **=** **1649)**		**Keeper (** ***n*** **=** **121)**		**Owner-keeper (** ***n*** **=** **1020)**	
Information	Modality	*n*	%	*n*	%	*n*	%	*n*	%
Professional activity related to equines								
	NC	48		26					
	No	609	89.6	1203	74.1				
	Yes	71	10.4	420	25.9				
	Total	728	100	1649	100				
Holding duration of equines (years)								
	NA					50		475	
	[0–5]					14	19.7	131	24.0
	[6–10]					22	31.0	122	22.4
	[10–20]					15	21.1	157	28.8
	Over 20					20	28.2	135	24.8
	Total					121	100	1020	100
Distance between owners and/or keepers and equines locations (km)^*^								
	0 (same commune)	126	17.8	1449	86.3	61	70.1	527	69.3
	[0–10]	198	27.9	93	5.5	7	8.0	130	17.1
	[10–20]	161	22.7	54	3.2	9	10.3	39	5.1
	[20–30]	75	10.6	23	1.4	1	1.1	13	1.7
	[30–40]	22	3.1	13	0.8	3	3.4	8	1.1
	[40–50]	20	2.8	9	0.5	1	1.1	5	0.7
	>50	107	15.1	38	2.3	5	5.7	39	5.1
	Total	709	100	1679	100	87	100	761	100

* *n corresponds to the number of lines but not to the number of owners and/or keepers because this value can have one or more holding communes. All percentages in this table were calculated without taking into account. NA, not applicable; NC, not considered*.

Most respondents to the owner survey (79%, *n* = 1812/2303) were nonprofessionals (professional activity not related to equines), and owner–keepers were more often professionals (professional activity related to equines) compared with owners (chi-squared test, *p* < 0.05) (Table 1).

Among the 616 keepers responding to the question on the year of starting to keep equines, most had been keepers for several years (Table 1).

### Keeping Habits

**Figure 2:**
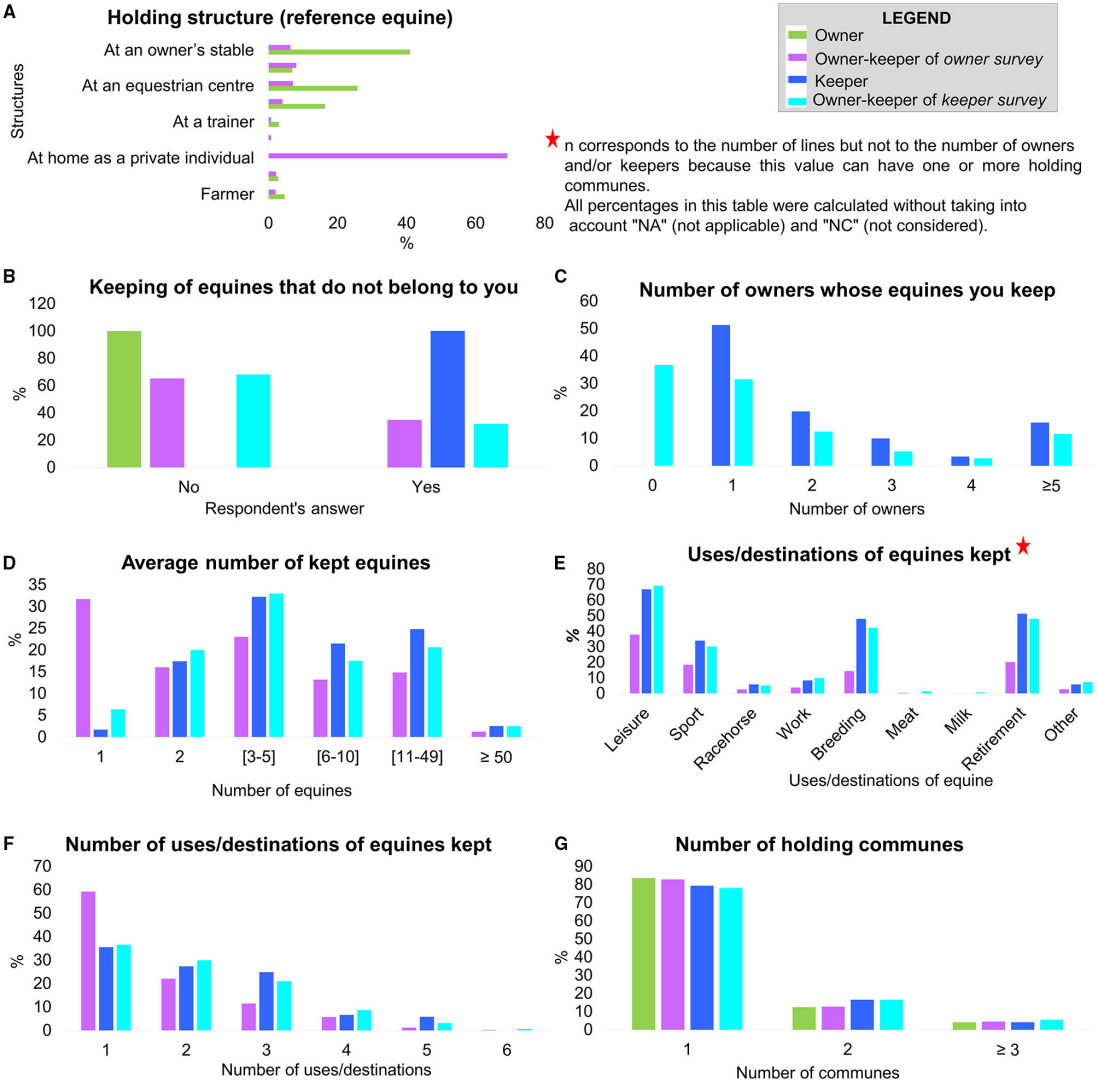
Detailed results of repartition the different keeping habits: repartition (percentage) per survey and type of respondent (owner, owner–keeper, and keeper).

The question on the holding structure concerned only respondents to the owner survey and the *reference equine*. The majority of owner–keepers kept their equine at home as private individuals (69%), and owners hosted their equine preferentially in a professional structure (41% in an owner's stable and 26% in an equestrian center). Most people who kept their equines at home were non-professionals (chi-squared test, *p* < 0.05) (Figure 2A).

Around one third of owner–keepers kept equines that did not belong to them (35%, *n* = 574/1649, in the owner survey and 32%, *n* = 325/1020, in the keeper survey) (Figure 2B). Nevertheless, equines kept came from a limited number of owners (Figure 2C) with proportions that differed according to the status (owner–keepers or keepers). The owner–keepers held equines from more than one owner compared with keepers (chi-squared test, *p* < 0.05).

Most people keeping equines kept a maximum of five equines (71%, *n* = 406/575, of owner–keepers from the owner survey; 59%, *n* = 606/1020, of owner–keepers from the keeper survey; and 51%, *n* = 62/121, of keepers) (Figure 2D, Supplementary Figure 1). Professional owner–keepers in the owner survey kept more equines than nonprofessionals (chi-squared test, *p* < 0.05). For the few respondents declaring that they kept more than 50 horses, the premises corresponded mainly to professional structures (stables, stud farms, or riding schools).

Most people kept one (43.9%, *n* = 750/1709) or two (27.2%, *n* = 465/1709) uses/destinations of equines together (Supplementary Table 1, Figure 2F). Around 20% (*n* = 310) of respondents kept equines with three use types. The most frequent uses/destinations of equines kept by respondents were leisure, retirement, breeding, and sport animals (Figure 2E). The two most frequent uses/destinations associated were leisure-retirement, leisure-breeding, leisure-sport, and sport-breeding (for more details see Supplementary Material Table 1).

Concerning the number of holding locations (evaluated on the basis of the number of *communes* with holdings), most respondents housed their equines in a single *commune* (81% (*n* = 2161/2669) of owner–keepers, 79% (*n* = 96/121) of keepers, and 83% (*n* = 608/728) of owners) (Figure 2G). The number of *communes* for equine premises varied significantly based on the number of equines kept regardless of the survey (Kruskal–Wallis test, *p* = 1.16^*^10^−18^ for owner–keepers from the owner survey, *p* = 1.72^*^10^−11^ for owner–keepers from the keeper survey, and *p* = 2.32^*^10^−12^ for keepers). As expected, the professional keepers had a significantly higher number of *communes* for equine premises than nonprofessionals (chi-squared test, *p* < 0.05).

### Relative Location of Equines, Owners, and Keepers

**Figure 3:**
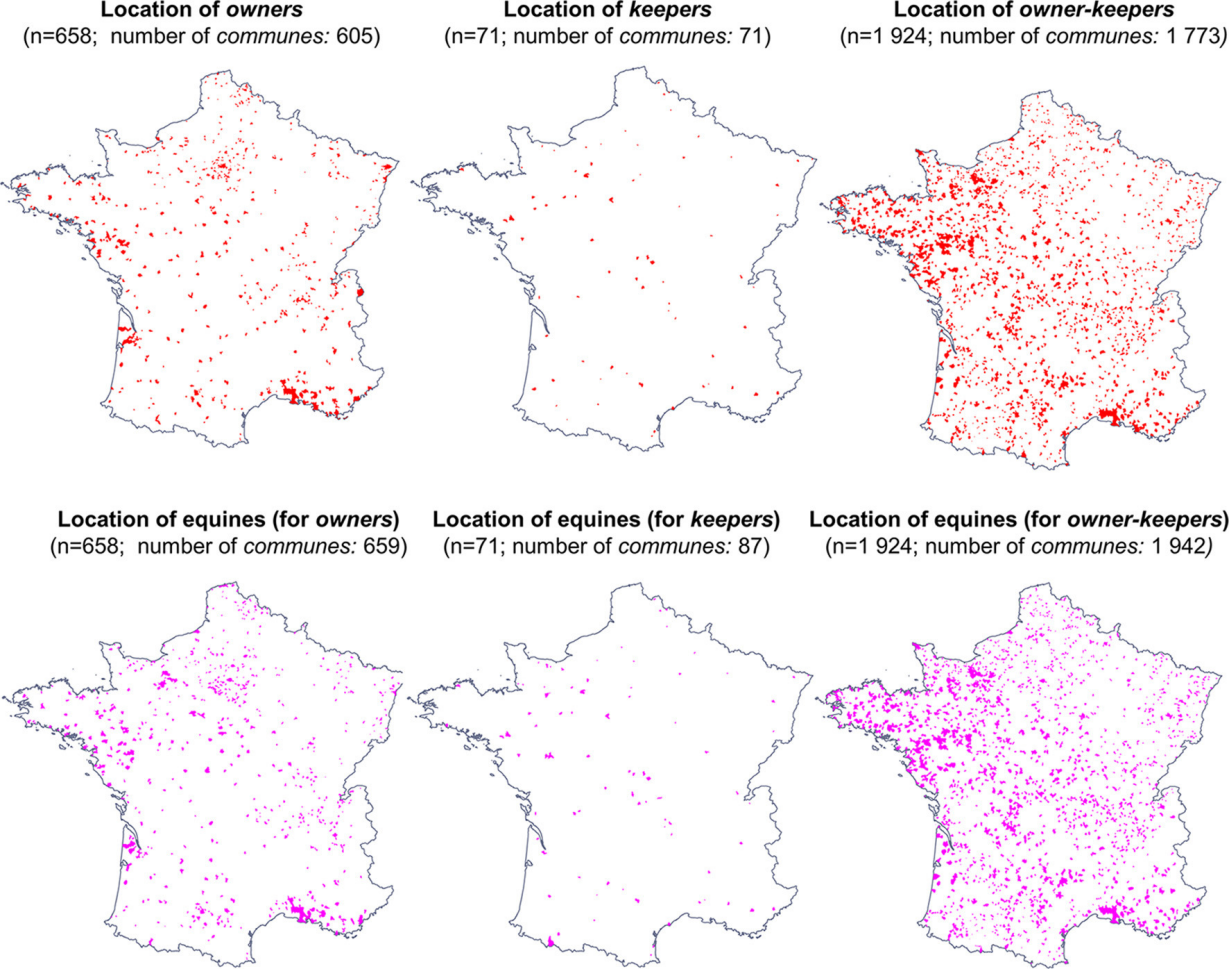
Residence and holding communes of respondents when the two communes were located in mainland France (a dot in a commune indicates that there is at least one owner, one keeper, one owner–keeper, or one equine in this commune).

Locations of people keeping equines and location of equines kept mainly overlapped regardless of whether equines belonged to them or not (86% of owners–keepers in the owner survey, 69% of owners–keepers in the keeper survey, and 70% of the only keepers). By contrast, only 18% of owners did not keep their equines housed in the same *commune* (Figure 3, Table 1).

When people and equines were located in different *communes*, most of them housed equines in *communes* fewer than 30 km away and very few over 50 km (Table 1). Median distance did not differ within surveys (Wilcoxon test, *p* > 0.05), but between the two surveys for owner–keepers (Wilcoxon test, *p* < 0.05). No correlation was found between the distance location and the level of urbanization of the *commune* of residence and neighboring communes.

## Discussion

This study provides additional data beyond the regular surveys lead by the IFCE. It is the first survey to reveal detailed information on keeping habits of equines in France including a majority of non-professionals. The habits of this population are currently not well known, but its weight in terms of numbers is important for the equine sector. From an epidemiological point of view, it may also play a significant role in the spread of equine diseases. As mentioned in a previous study (3), the main limitation of these surveys is that, among all owners (*n* = 737,789) and keepers (*n* = 76,501) recorded in the SIRE database, very few were contactable (1.9% of owners and 8.2% of keepers) and additionally responded to the survey (at last 0.4% of owners and 1.6% of keepers recorded in the SIRE database). This implies that respondents to the survey may not represent all owner and keepership profiles, for instance, occasional owner–keepers, who may be the least informed and most difficult to inform regarding their legal obligation and also to contact in case of an epidemic. Another limitation is that certain questions were asked only in one survey, and also for a specific equine, which led to incomplete information, such as a lack of information on the type of holding and degree of professionalization of keepers.

Our study revealed various keeping habits depending on the profiles of the respondent owners, owner–keepers, or keepers and confirmed the complexity of the equine sector, linked to the mix of professionals and privates. When modeling the spread of diseases or setting up surveillance methods, this factor of variation should be taken into account as the three categories cannot be managed in the same way.

In our study, broad geographic dispersion of equines was observed with most of them being held in small structures (fewer than five equines kept together) by private individuals. Our results relating to keeping habits are not in agreement with the previous study by Vial et al. (6), which predicted a positive relationship between the number of equines kept and “home-keeping”. This could be due to the difference in the studied population (comprehensive local censuses *vs*. national partial survey) and also the more complex determinants relating to professional structures and/or land availability (7). In the same way, our results provide a different perspective from that of the annual surveys conducted by the IFCE (8). Indeed, our work addressed owners and keepers, and IFCE targets equines in its surveys. This explains differences observed in our results with 57% of people housing their equine(s) at home as a private individual (declared or not), 1.5% in training premises, and 12.4% in an equestrian center although surveys of the IFCE indicates 30% of equines kept in individual private and declared structures, 4% in training premises, and 32% in an equestrian center. Additionally, the definition of professional or non-professional was declarative in our study, and the IFCE considers as professional anyone who has a declared agricultural activity even if it is minor. This fragmentation of non-professional owner–keepers and their equines (not necessarily kept together) is a real challenge for providing information in the event of an outbreak and also a major difficulty in terms of monitoring and isolating this population during an epidemic. This specific characteristic must be taken into account during epidemiological investigations and also in research when modeling disease spread and control measure effects.

Our study indicates a low connection of owner–keepers and keepers with a small number of equines kept from a limited number of owners. In that way, this population is distinct from the highly connected population encountered in professional structures (breeding, barns, equestrian centers). Nevertheless, our study did not allow estimating the indirect contact between people and/or equines, such as pasture and local leisure contact as well as limited local movements (gathering, collective hiking…). These factors are important for local and slow dissemination of contact diseases such as strangles (9), vector-transmitted diseases such as equine infectious anemia (10), or soil-borne diseases such as anthrax (11). This apparent less connected population could also play a more important role than expected, especially because it is the most difficult to reach.

Knowledge of the types and associations of uses/destinations of equines is important to understand the organization of this non-professional part of the equine sector (6, 7). Consistent with the work of Vial at al. (6), our study shows that owners keeping leisure or retirement equines were used to managing them themselves, this kind of equine being part of the family and their care and use not requiring a specific structure or equipment. It is also an important factor to include when predicting the frequency and distances of equine movements and thereby the risk of contamination or the spread of disease. This knowledge is useful for epidemiological modeling and also for disease surveillance. In our study, few of the respondents kept racehorses (Supplementary Figure 2), yet these horses move around extensively, often over long distances, and can be a source of diseases spreading locally and also at long distances during equine gatherings for races. Nevertheless, the low level of interaction of this at-risk population with other equine populations limits the risk in terms of disease spread, especially because racehorses are closely monitored by the horseracing authorities in France.

Regarding distances, most equines in our study lived near to their owners and/or keepers. This was consistent with the results of studies of Vial et al. (6, 7), an expected result given that respondents were mainly individuals caring for their equines themselves, which requires having them at a distance compatible with daily care visits. We obtained similar results to those previously found in Great Britain (12, 13). In their studies, Robin et al. found that most owners lived close to their equines. In these studies, 61% of equines (in one) and 53% (in the other) lived at the owner's location vs. 66% in our study. The distance was >50 km for only 2 and 5% of equines, respectively, vs. 6.1% in our study. In addition, about 90% of equines were fewer than 10 km from their owners vs. 78.1% in our case. However, the degree of precision of the location in the British study and in our study probably differs, and this could lead to artificial similarity. In Great Britain, the distances were probably more accurate geographically because the studies used postcodes, generally assigning a location to a very precise area (each postcode is valid for a street, part of a street, or a set of houses, at least in urban areas). By contrast, *communes* in France are larger, and calculated distances probably did not reflect the exact situation because we used the centroid of the *commune* (the only information available). However, the geographic area covered by a *commune* contains several plausible localization points depending on land use. Equines and owners located in the same *commune* may be far from one another or very near. When equines are in different *communes*, they could be located close to the administrative border and, thus, be very close to their owners/keepers in actual geographic terms. The use of exact addresses would have been effective for accurate localization of equines as well as their owners, but the EU General Data Protection Regulation (14) makes it difficult to obtain and use such information. This lack of precision concerning distances may explain why no, in the global sample, correlation between these distances and the rate of urbanization was found but that a correlation was only observed for a subsample of owners and owner–keepers of the owner survey. In Great Britain, an inverse correlation between built-up land use and the proportion of horses kept at the same postcode as the owner's address was detected (13). This could be explained by the cultural differences related to equines between the two countries, for instance, greater familiarity with and knowledge of equines in England, whereby owners may have more facilities to keep their equines themselves. The location of the owner can provide a useful indication of the spatial distribution of equines and may depend on the uses/destinations of equines (6, 7). However, other additional information can and should be taken into account to better understand factors influencing the location of equines and also to credibly estimate their spatial distribution. The CLC database could provide new insights as it contains detailed information on land use in 39 European states, including France. Likewise, the Fallen Stock Data Interchange database could provide the required additional information, in the form of the location of equines dying in France (15, 16).

## Conclusions

This study provided information of great interest for the equine sector, in particular concerning small private owners and keepers. Knowledge of the target population is an important aspect to properly inform and involve it and also to manage health events or disease outbreaks and to adapt surveillance and control measures to limit the impact of these events. In addition, this study provided a highly valuable sample of owners–equines with precise localization and detailed characteristics. This sample is being used, in association with other additional data sources, to obtain a realistic estimation of the location of equines in France.

## Data Availability Statement

The raw data supporting the conclusions of this article will be made available by the authors, without undue reservation.

## Author Contributions

JT, CS, and MS designed the study. JT, CS, AM, MS, XD, and MD contributed to the conception of the questionnaire. HF, AM, XD, and MD contributed to the data collection. HF, JT, CS, and DG contributed to the data analysis. HF wrote the manuscript with support from CS, JT, and DG. All authors participated to the interpretation of the results.

## Conflict of Interest

The authors declare that the research was conducted in the absence of any commercial or financial relationships that could be construed as a potential conflict of interest.

## Publisher's Note

All claims expressed in this article are solely those of the authors and do not necessarily represent those of their affiliated organizations, or those of the publisher, the editors and the reviewers. Any product that may be evaluated in this article, or claim that may be made by its manufacturer, is not guaranteed or endorsed by the publisher.
