# Supplementary material for: Home Sweet Home: New Insights Into the Location of Equine Premises in France and Keeping Habits to Inform Health Prevention and Disease Surveillance
Source: Front Vet Sci. 2021 Aug 23;8:701749. doi: 10.3389/fvets.2021.701749 (PMC8419474; doi:10.3389/fvets.2021.701749)
Supplement: Supplementary file 1 [file Table_1.DOCX]

Table S1 : Uses/destinations of equines kept

|  |  | Owner survey | | | Keeper survey | | | |
| --- | --- | --- | --- | --- | --- | --- | --- | --- |
|  |  | *Owners* | *Owner-keepers* | | *Keepers* | | *Owner-keepers* | |
|  |  | n | n | % | n | % | n | % |
| 1 use/destination | Leisure |  | 178 | 52.4 | 23 | 53.5 | 229 | 62.4 |
|  | Sport |  | 33 | 9.7 | 2 | 4.7 | 16 | 4.4 |
|  | Racehorse |  | 11 | 3.2 | 0 | 0 | 3 | 0.8 |
|  | Work |  | 3 | 0.9 | 0 | 0 | 3 | 0.8 |
|  | Breeding |  | 38 | 11.2 | 11 | 25.6 | 39 | 10.6 |
|  | Meat |  | 0 | 0 | 0 | 0 | 1 | 0.3 |
|  | Milk |  | 0 | 0 | 0 | 0 | 0 | 0.0 |
|  | Retirement |  | 64 | 18.8 | 7 | 16.3 | 64 | 17.4 |
|  | Other |  | 13 | 3.8 | 0 | 0 | 12 | 3.3 |
|  | **Total** |  | **340** | **100** | **43** | **100** | **367** | **100** |
| 2 uses/destinations | Racehorse, breeding |  | 5 | 3.9 | 2 | 6.1 | 11 | 3.6 |
|  | Racehorse, retirement |  | 0 | 0.0 | 0 | 0 | 1 | 0.3 |
|  | Breeding, retirement |  | 8 | 6.3 | 2 | 6.1 | 24 | 7.9 |
|  | Breeding, other |  | 0 | 0 | 0 | 0 | 3 | 1.0 |
|  | Breeding, meat |  | 0 | 0 | 0 | 0 | 4 | 1.3 |
|  | Leisure, other |  | 4 | 3.1 | 1 | 3.0 | 9 | 3.0 |
|  | Leisure, breeding |  | 4 | 3.1 | 3 | 9.1 | 47 | 15.4 |
|  | Leisure, retirement |  | 45 | 35.4 | 11 | 33.3 | 108 | 35.4 |
|  | Leisure, sport |  | 33 | 26.0 | 4 | 12.1 | 27 | 8.9 |
|  | Leisure, work |  | 4 | 3.1 | 2 | 6.1 | 9 | 3.0 |
|  | Retirement, other |  | 1 | 0.8 | 1 | 3.0 | 5 | 1.6 |
|  | Sport, breeding |  | 17 | 13.4 | 4 | 12.1 | 32 | 10.5 |
|  | Sport, retirement |  | 5 | 3.9 | 2 | 6.1 | 18 | 5.9 |
|  | Sport, other |  | 0 | 0 | 0 | 0 | 1 | 0.3 |
|  | Work, retirement |  | 1 | 0.8 | 1 | 3.0 | 2 | 0.7 |
|  | Work, other |  | 0 | 0 | 0 | 0 | 1 | 0.3 |
|  | Work, breeding |  | 0 | 0 | 0 | 0 | 3 | 1.0 |
|  | **Total** |  | **127** | **100** | **33** | **100** | **305** | **100** |
| 3 uses/destinations | Racehorse, breeding, retirement |  | 0 | 0 | 0 | 0 | 11 | 5.1 |
|  | Racehorse, breeding, meat |  | 1 | 1.5 | 0 | 0 | 1 | 0.5 |
|  | Racehorse, work, breeding |  | 1 | 1.5 | 0 | 0 | 2 | 0.9 |
|  | Breeding, retirement, other |  | 0 | 0 | 0 | 0 | 4 | 1.9 |
|  | Leisure, racehorse, retirement |  | 1 | 1.5 | 2 | 6.7 | 3 | 1.4 |
|  | Leisure, breeding, retirement |  | 7 | 10.6 | 9 | 30.0 | 53 | 24.8 |
|  | Leisure, sport, racehorse |  | 1 | 1.5 | 1 | 3.3 | 1 | 0.5 |
|  | Leisure, sport, breeding |  | 14 | 21.2 | 5 | 16.7 | 33 | 15.4 |
|  | Leisure, sport, retirement |  | 20 | 30.3 | 6 | 20.0 | 37 | 17.3 |
|  | Leisure, work, breeding |  | 3 | 4.5 | 1 | 3.3 | 7 | 3.3 |
|  | Leisure, racehorse, breeding |  | 0 | 0 | 0 | 0 | 4 | 1.9 |
|  | Leisure, breeding, other |  | 0 | 0 | 0 | 0 | 1 | 0.5 |
|  | Leisure, breeding, meat |  | 0 | 0 | 0 | 0 | 1 | 0.5 |
|  | Leisure, retirement, other |  | 0 | 0 | 0 | 0 | 7 | 3.3 |
|  | Leisure, sport, other |  | 1 | 1.5 | 0 | 0 | 2 | 0.9 |
|  | Leisure, sport, meat |  | 1 | 1.5 | 0 | 0 | 0 | 0 |
|  | Leisure, sport, work |  | 6 | 9.1 | 0 | 0 | 5 | 2.3 |
|  | Leisure, work, other |  | 0 | 0.0 | 0 | 0 | 1 | 0.5 |
|  | Leisure, work, retirement |  | 1 | 1.5 | 0 | 0 | 6 | 2.8 |
|  | Sport, racehorse, breeding |  | 0 | 0 | 0 | 0 | 1 | 0.5 |
|  | Sport, breeding, retirement |  | 0 | 0 | 0 | 0 | 22 | 10.3 |
|  | Sport, retirement, other |  | 3 | 4.5 | 0 | 0 | 1 | 0.5 |
|  | Sport, work, other |  | 0 | 0 | 0 | 0 | 1 | 0.5 |
|  | Sport, work, breeding |  | 1 | 1.5 | 0 | 0 | 2 | 0.9 |
|  | Sport, breeding, retirement |  | 4 | 6.1 | 6 | 20.0 | 0 | 0 |
|  | Sport, work, meat |  | 1 | 1.5 | 0 | 0 | 0 | 0 |
|  | Work, breeding, retirement |  | 0 | 0 | 0 | 0 | 6 | 2.8 |
|  | Work, breeding, meat |  | 0 | 0 | 0 | 0 | 1 | 0.5 |
|  | Work, meat, milk |  | 0 | 0 | 0 | 0 | 1 | 0.5 |
|  | **Total** |  | **66** | **100** | **30** | **100** | **214** | **100** |
| 4 uses/destinations | Leisure, racehorse, breeding, retirement |  | 0 | 0 | 1 | 12.5 | 2 | 2.2 |
|  | Leisure, sport, breeding, retirement |  | 21 | 63.6 | 3 | 37.5 | 49 | 55.1 |
|  | Leisure, sport, racehorse, breeding |  | 0 | 0 | 0 | 0 | 3 | 3.4 |
|  | Leisure, sport, milk, retirement |  | 0 | 0 | 0 | 0 | 2 | 2.2 |
|  | Leisure, sport, work, other |  | 0 | 0 | 0 | 0 | 1 | 1.1 |
|  | Leisure, sport, work, breeding |  | 4 | 12.1 | 0 | 0 | 2 | 2.2 |
|  | Leisure, sport, work, retirement |  | 4 | 12.1 | 0 | 0 | 5 | 5.6 |
|  | Leisure, sport, breeding, other |  | 2 | 6.1 | 0 | 0 | 0 | 0 |
|  | Leisure, breeding, retirement, other |  | 0 | 0 | 2 | 25 | 5 | 5.6 |
|  | Leisure, work, breeding, retirement |  | 0 | 0 | 1 | 12.5 | 8 | 9.0 |
|  | Leisure, work, retirement, other |  | 0 | 0 | 0 | 0 | 1 | 1.1 |
|  | Sport, racehorse, breeding, retirement |  | 0 | 0 | 0 | 0 | 1 | 1.1 |
|  | Sport, racehorse, work, breeding |  | 0 | 0 | 0 | 0 | 1 | 1.1 |
|  | Sport, breeding, retirement, other |  | 0 | 0 | 0 | 0 | 3 | 3.4 |
|  | Sport, breeding, meat, retirement |  | 0 | 0 | 0 | 0 | 1 | 1.1 |
|  | Sport, work, breeding, other |  | 1 | 3.0 | 0 | 0 | 1 | 1.1 |
|  | Sport, work, breeding, retirement |  | 1 | 3.0 | 0 | 0 | 3 | 3.4 |
|  | Sport, breeding, retirement, other |  | 0 | 0 | 1 | 12.5 | 0 | 0 |
|  | Work, breeding, retirement, other |  | 0 | 0 | 0 | 0 | 1 | 1.1 |
|  | **Total** |  | **33** | **100** | **8** | **100** | **89** | **100** |
| 5 uses/destinations | Leisure, sport, breeding, retirement, other |  | 0 | 0 | 1 | 14.3 | 8 | 25 |
|  | Leisure, sport, work, breeding, retirement |  | 3 | 42.9 | 5 | 71.4 | 16 | 50 |
|  | sport, racehorse, breeding, retirement, other |  | 0 | 0 | 1 | 14.3 | 0 | 0 |
|  | Leisure, sport, racehorse, breeding, retirement |  | 2 | 28.6 | 0 | 0 | 2 | 6.3 |
|  | Leisure, sport, racehorse, work, breeding |  | 0 | 0 | 0 | 0 | 1 | 3.1 |
|  | Leisure, sport, breeding, meat, retirement |  | 0 | 0 | 0 | 0 | 1 | 3.1 |
|  | Leisure, sport, work, retirement, other |  | 0 | 0 | 0 | 0 | 1 | 3.1 |
|  | Leisure, work, breeding, milk, retirement |  | 0 | 0 | 0 | 0 | 1 | 3.1 |
|  | Leisure, work, breeding, meat, milk |  | 0 | 0 | 0 | 0 | 1 | 3.1 |
|  | Leisure, work, breeding, retirement, other |  | 1 | 14.3 | 0 | 0 | 0 | 0 |
|  | Leisure, sport, racehorse, work, retirement |  | 1 | 14.3 | 0 | 0 | 0 | 0 |
|  | Sport, racehorse, work, breeding, retirement |  | 0 | 0 | 0 | 0 | 1 | 3.1 |
|  | **Total** |  | **7** | **100** | **7** | **100** | **32** | **100** |
| 6 uses/destinations | Leisure, sport, racehorse, work, breeding, retirement |  | 1 | 100 | 0 | 0 | 2 | 28.6 |
|  | Leisure, sport, work, breeding, retirement, other |  | 0 | 0 | 0 | 0 | 3 | 42.9 |
|  | Leisure, work, breeding, meat, milk, retirement |  | 0 | 0 | 0 | 0 | 2 | 28.6 |
|  | **Total** |  | **1** | **100** | **0** | **0** | **7** | **100** |
