# Supplementary figures and images for: Home Sweet Home: New Insights Into the Location of Equine Premises in France and Keeping Habits to Inform Health Prevention and Disease Surveillance
Source: Front Vet Sci. 2021 Aug 23;8:701749. doi: 10.3389/fvets.2021.701749 (PMC8419474; doi:10.3389/fvets.2021.701749)

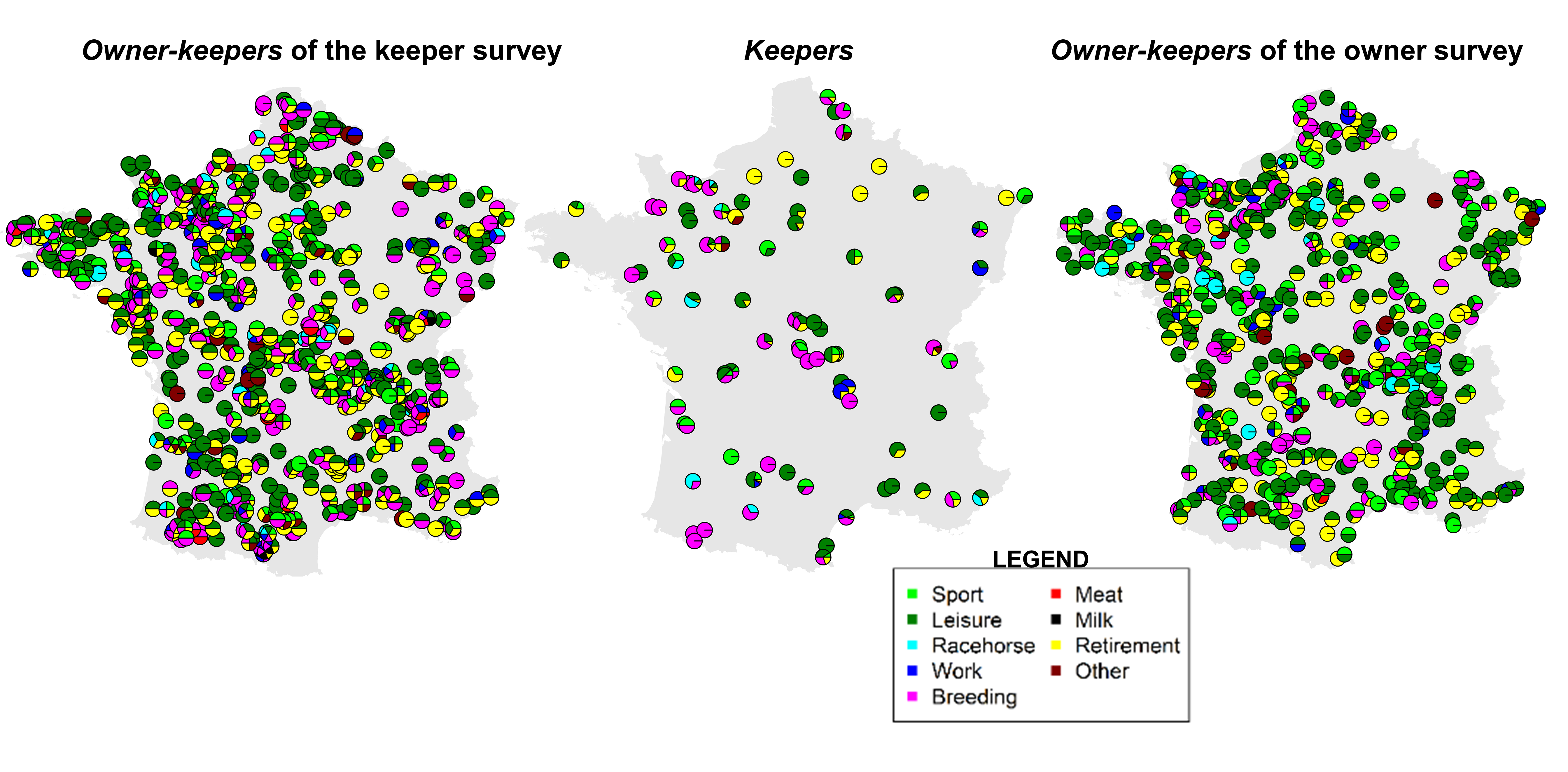

Supplement: Supplementary file 3 [file Image_2.TIF]
